# Extracellular vesicles (exosomes and ectosomes) play key roles in the pathology of brain diseases

**DOI:** 10.1186/s43556-021-00040-5

**Published:** 2021-06-20

**Authors:** Jacopo Meldolesi

**Affiliations:** Division of Neuroscience, San Raffaele Institute and Vita-Salute San Raffaele University, via Olgettina 58, 20132 Milan, Italy

**Keywords:** Neurons, Astrocytes, Microglia, Immunological and neurodegenerative diseases, Multiple sclerosis, Alzheimer’s disease

## Abstract

Last century, neurons and glial cells were mostly believed to play distinct functions, relevant for the brain. Progressively, however, it became clear that neurons, astrocytes and microglia co-operate intensely with each other by release/binding of signaling factors, direct surface binding and generation/release of extracellular vesicles, the exosomes and ectosomes, called together vesicles in this abstract. The present review is focused on these vesicles, fundamental in various brain diseases. Their properties are extraordinary. The specificity of their membrane governs their fusion with distinct target cells, variable depending on the state and specificity of their cells of origin and target. Result of vesicle fusion is the discharge of their cargos into the cytoplasm of target cells. Cargos are composed of critical molecules, from proteins (various nature and function) to nucleotides (especially miRNAs), playing critical roles in immune and neurodegenerative diseases. Among immune diseases is multiple sclerosis, affected by extensive dysregulation of co-trafficking neural and glial vesicles, with distinct miRNAs inducing severe or reducing effects. The vesicle-dependent differences between progressive and relapsing-remitting forms of the disease are relevant for clinical developments. In Alzheimer’s disease the vesicles can affect the brain by changing their generation and inducing co-release of effective proteins, such Aβ and tau, from neurons and astrocytes. Specific miRNAs can delay the long-term development of the disease. Upon their traffic through the blood-brainbarrier, vesicles of various origin reach fluids where they are essential for the identification of biomarkers, important for diagnostic and therapeutic innovations, critical for the future of many brain patients.

## Introduction

**Fig. 1:**
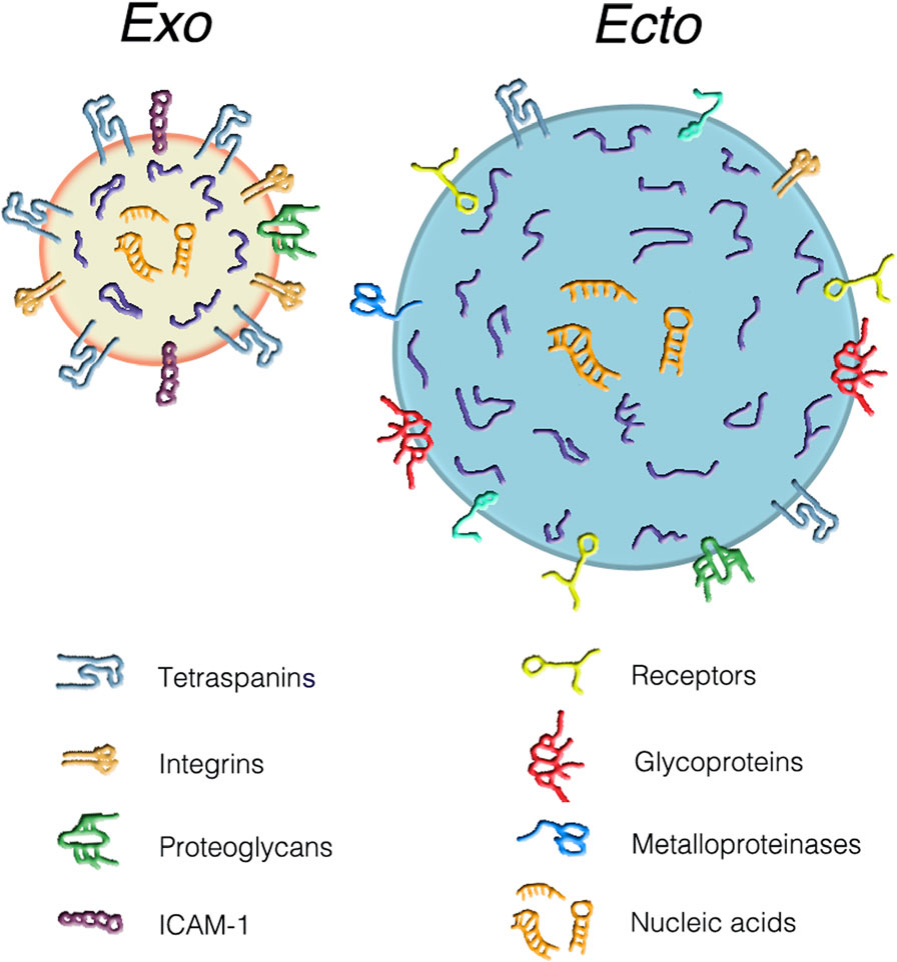
The two types of EVs: exosomes (left) and ectosomes (right). The figure shows the two types of EVs. In terms of size the exosomes (gold content) are smaller (diameters of 50–150 nm) while the ectosomes (sky-blue content) are larger (diameter of 150–500 nm). In terms of composition the membranes of exosomes are rich in tetraspanins, a protein complex highly important for the distribution of other proteins including those trapped at the luminal surface. A lower density of tetraspanins is present in the membrane of ectosomes. A similar partial difference is true for integrins and proteoglycans. In contrast, the adhesion molecule 1 (ICAM-1), is appreciable only in the exosome membrane. The ectosome membrane is rich in other proteins: receptors, glycoproteins, metalloproteinases and others. Among the EV membrane proteins, some of the cytosol establish specific binding with surface receptors of target cells, a process necessary for the subsequent EV uptake. The lumenal cargos are similar in the two EV types. They contain many typical proteins (blue strings), some of which anchored to the EV membrane, mixed with low concentrations of cytosolic proteins. The lumena of both EVs show various types of orange sequences composed by nucleic acids, i.e. the coding mRNAs, the non-coding miRNAs and cicrRNAs and, in some cases, also DNA sequences. Reproduced with permission from Fig. 2 of Ref. 2

**Fig. 2:**
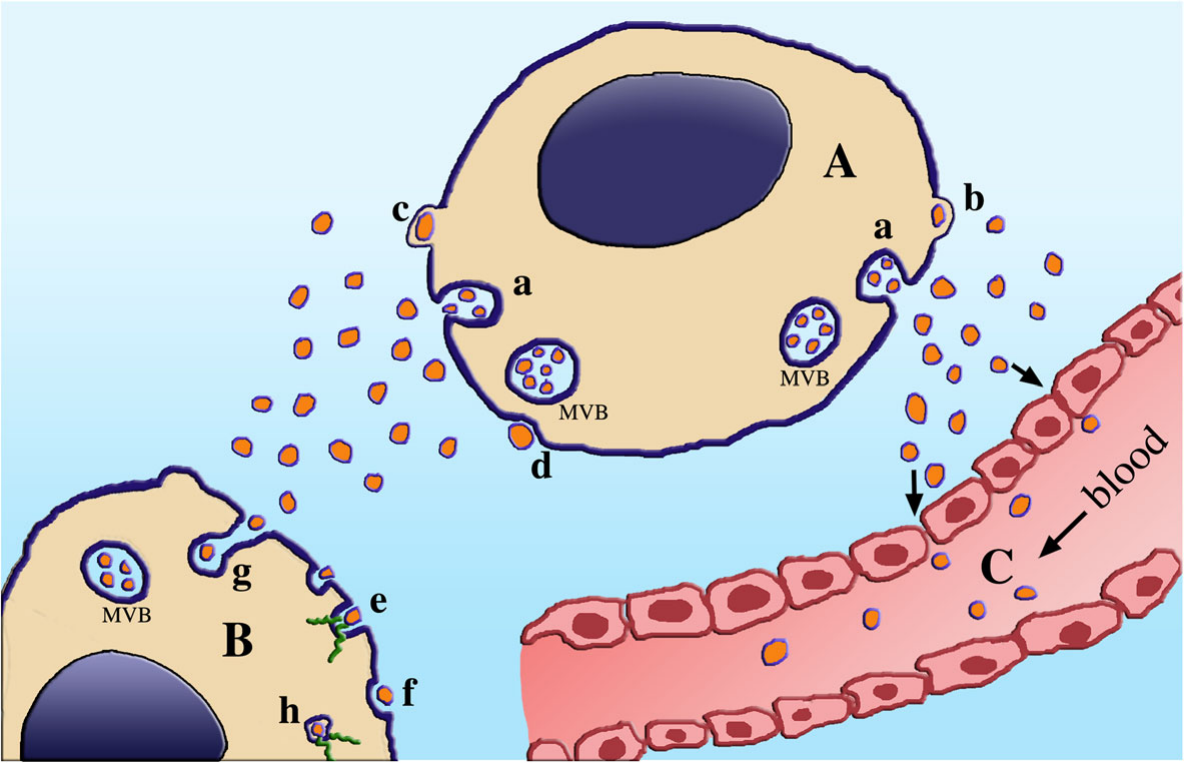
Secretion of EVs by the cell A, their navigation in the extracellular space, and their uptake by the target cell B and a blood capillary C. Secretion is shown in the upper cell (A, gray color). Exosomes (small, red), previously accumulated within multi-vesicular bodies (MVB) of endocytic nature, are released upon the exocytosis of the latter (a) [1]. Ectosomes are secreted by a completely different process: upon their assembly and growth at the cytosolic surface of peculiar rafts continuous to the plasma membrane (b,c) they are converted into EVs released from the cell surface by pinching off and then shedding of the rafts (d). As it occurs within tissues, the EVs navigating in the extracellular space are addressed to various targets (two in this Figure). The cell to the left (B) receives EVs released from the left side of cell A. Upon their association by receptor binding to the surface of cell B, the EVs disassemble their cargos which are transferred to the cytoplasm along green short lines (e); for other EVs the process is analogous, however it occurs after their endocytic uptake and internalization (g,h). The EVs to the right are taken up into a large blood capillary lumen (C, red pink color), reached upon their transfer first across the blood-brainbarrier (not shown) and then through the junctions between endocytic cells, pointed here by arrows

The system of extracellular vesicles (EVs) includes two types of mini-structure, the exosomes, generated and then released by multivesicular bodies (MVBs), a specialized vacuole of the endosomal system; and the larger ectosomes (also called microvesicles), generated and released by the plasma membrane (Figs. 1 and 2). Specific for their membrane and the cargos of their lumen, EVs cover many critical functions of traffic, transport to and exchange among cells. Mentioned first in the nineteen eighties as vesicles released by reticulocytes during maturation [3], the EVs were more widely reported in the following decade and then characterized in a few, distinct types of cells [4, 5]. Subsequent studies clarified that the EV system is a property of many, possibly of all types of cells. The vesicles, heterogeneous and complex in structure, are mostly molecularly distinct from the membranes and cytosol from which they originate [6, 7]. While the membranes play key roles for interactions and fusions, the distinct luminal cargos, protected from extracellular degradation, are finally discharged into the cytoplasm of target cells (Fig. 2). Cargos are highly variable. They do not include only proteins, but also lipids, proteolipids, carbohydrates and also nucleotides (Fig. 1). Cargo RNAs, abundant in most EVs, include coding (mRNAs) and non-coding (miRNAs, cicrRNAs) forms, critical in cell physiology and pathology [8, 9]. In addition, EVs participate in other cell functions, including the generation of diagnostic biomarkers and the development of therapeutic innovations [10–12].

The present review deals with the EV system of the brain, focusing on three types of cells: neurons, astrocytes and microglia. Compared to other organs, the discovery of brain vesicles has been delayed. Conclusive demonstration of brain EVs was first reported at the beginning of the last decade [13]. Since then, the frequency of EVs increased progressively, reaching its top in 2019–2020 [14–16]. Initially, the delay of the EV brain studies was due primarily to the complexity of the organ, the heterogeneity (compositional, structural and functional) of the different cell types and areas, the variable ratios between neurons and the various types of glial cells [17–19]. These and possibly other properties of the brain appeared problematical for the isolation and analysis of the various types of EVs. However, improvements have been developed. At present, therefore, the various brain EVs are much better understood [18, 19]. Their total weight has been calculated to approach the 10% of the total weight of brain cells [20].

In terms of traffic, EVs share properties with viruses [12], i.e. they move across the blood-brainbarrier with ensuing distribution not only in the space around the cells, but also in the circulating fluids of cerebrospinal fluid (CSF), blood serum (Fig. 2) and others. Based on these considerations it became rapidly clear the chance to investigate brain EVs, not only when isolated from the organ or its various cells, but also from biological fluids. Numerous results about fluids, including the isolation of specific EVs, have been reported [21–28]. All together the fluid results were highly encouraging because they reveal differences between controls and patients, especially those of neurodegenerative diseases [21–24, 26]; and because some of the fluid EV data were found to resemble the corresponding vesicles isolated from brain tissue [26, 27].

In conclusion, for quite some time the studies of brain EVs, compared to the EVs of other organs and tissues, were problematical. Progressively, however, such problems have been first diluted and then solved. At the moment, life of EVs is well known from their generation followed by secretion, traffic and fusion to specific types of target cells (Fig. 2). In the following areas I first anticipate the EV events relevant in the two general states of brain cells, physiology and pathology. The following two Sections are dedicated to immunological and neurodegenerative diseases. Interestingly, the two families of diseases are not always distinct. Properties of both of them, in fact, are present in single diseases, such as amyotrophic lateral sclerosis (ALS) and other diseases.

## Role of EVs during brain physiology and pathology

Critical functions of EVs take place both during the development and upon maturation of brain cells. Until recently, physiological processes were attributed to single types of brain cells and their intracellular organelles. Now the frequent participation of two, and even three types of cells, with secretion of both types of EVs (Fig. 2), is generally accepted. Additional processes dependent on EVs take place in the course of diseases, i.e. EVs are relevant also in pathology.

EVs are involved in many functions, focused not only on whole cells but also on their portions, for example neuronal synapses (development, regulation, strength) and axons (growth, regeneration) [17]. The various steps of EV generation (establishment of their specific membranes; accumulation and assembly of their cargos; their secretion to the extracellular space) take place in response to cell stimulation. In addition to the proteins, lipids, carbohydrates and various RNAs (Fig. 1), the EV cargos include proteins of intracellular organelles such as mitochondria and lysosomes, as well as functional agents such as cytokines, chemokines, growth factors, other peptides and eicosanoids, neurotoxins, amyloid-β (Aβ), tau and other disease agents. These components remain segregated until the EV fusion results in the discharge of their cargos, most often into the cytoplasm of target cells (Fig. 2) [17–19, 29].

### Role of brain EVs in physiology

The role of EVs is relevant since it contributes to the protection and survival of cells, in particular of neurons, the most risky cells of the brain tissue. The first step considered is the role of neurons that secrete EVs destined to target other neuronal cells. The results confirmed the critical role of neuronal EVs. They, in fact, operate in important processes such the regulation of neurogenesis and the assembly of neuronal circuits [30]. Additional evidence of considerable importance, dependent on inter-neuronal EVs, emerged from results of cell plasticity, concerning in particular the physiology of synapses. In this case, the development induced by appropriate EVs and the negative control induced by their excess, take place in excitatory glutamatergic synapses [31]. Likewise, secretion of EVs from distinct neurons, one including the tyrosine kinase receptor Eph, the other the ligand ephrin, participate in the axonal growth and synaptic physiology [32].

Another main step of brain physiology is due to the new role of microglia. For many decades, the functional role of microglia, independent from their interactions with neurons and astrocytes, was believed to include only homeostasis. The progress of the last 20 years has demonstrated that homeostatic microglia includes also a number of reactive functions. Such conversion, largely related to pro-inflammatory responses, depends on the activation of an important surface receptor, TREM2. Therefore, the actions of microglia, and those of their EVs, occur not only in pathology but also in the course of physiological processes [33, 34]. The role of microglia, sustained by the interaction of their EVs with the other brain cells, induces therefore important initiatives, until recently unexpected. An example: active endocannabinoids, traditionally considered of neuronal postsynaptic nature, have been shown to originate from microglia. Their EVs are addressed to the presynaptic area of neurons, where GABAergic activity is reduced [35].

A key role of microglia concerns inflammation. The EVs involved are often enriched in miRNAs, critical tools that regulate the activity of target cells [11, 19, 21, 24, 25, 27]. In these cases the microglia EVs have been shown to modulate the functional activity of their target neurons [19, 36]. Analogous results have been obtained by other EVs of microglial origin, enriched however not in miRNAs but in another form of non-coding RNA, the Y-RNA. Even if operative mechanisms are still unclear, the effects of these RNAs have been shown to reduce neuronal activity [37].

An additional, important role in the brain concerns EV secretion by astrocytes. In this case the cooperation occurring between neurons and glial cells include development and possible elimination of synapses [38, 39]. The EVs from astrocytes are often rich of miRNAs. When EVs of this origin, enriched in the non-coding RNA miR-7, are targeted to neurons, the activity of the latter is decreased, especially at the level of synapses [40]. Another main component of astrocyte-derived EVs, miR-873a-5p, is known to attenuate a microglia-mediated neuroinflammation. In this case, the final effect, an improvement of a neurological defect, is due to a reduction of microglia EV transfer [41]. In an additional study the effects of astrocytes to neurons are numerous, often protective but in some cases also inhibitory. Such effects, however, take place without mediation [39]. In conclusion, many brain physiological events are due to EV interactions between neurons and glial cells.

### Role of brain EVs in pathology

Various processes, already considered among physiological properties, are relevant also for changes developed during specific diseases. Among the processes to be considered are the communications established by EVs between neurons and glial cells [19, 38–40]. Of interest in these cases are the general overviews for clinical practice [10] including the identification of biomarkers and the development of promising therapeutic approaches [11, 23]. Appropriate EVs participate also in the extension of functional contacts, such as the binding of agents to their specific receptors [32–34, 42]. These communications can contribute to the development of learning and memory [43].

The relevance of EV communications between neurons and glial cells often depends on the different miRNA involved. The profiles and levels of the nucleotides within EVs involved in neurodegenerative diseases, such as Parkinson’s and disease (PD) and Alzheimer’s disease (AD), are different from those secreted by corresponding physiological cells [21, 22, 24]. Interestingly, two types of miRNA, operative in EVs from neurons targeted to microglia, appear cell protective during brain traumatic injuries and hemorrhages [44]. Additional aspects of miRNAs from EVs refer also to medical practice in various diseases. One such perspective concerns the development of biomarkers, special molecules relevant for the diagnosis of diseases. Specific EVs appear as promising sources for their recognition, operative by ensuing opportunities and challenges [21–23, 25],

An additional relevant role of EVs refers to new mechanisms of specific therapy. Examples are miRNAs employed in potential clinical interventions [10] and EVs derived from immune cells [45]. In mechanistic terms, the therapeutic potential of managed EVs is of interest. Upon preventive simplification of their cargos, with elimination of unnecessary components, the EVs can accumulate drugs or factors underpinning a range of processes by therapeutic potential. The EV surface should be appropriately designed, addressed to target cells where their cargos (including drugs and factors) should be delivered. New methods are being developed to improve and engineer EVs to tailor the desired therapeutic factors to outcome [19, 28]. Depending on the diseases, the requests can vary. As an example, see the complex strategy of antiviral therapy [12].

## Immunological diseases of the brain

The role in brain physiology and pathology of EVs from neural and glial cells has already been discussed. Here, I will introduce data about brain immunological diseases. Among glial cells, the most important for inflammation is microglia, able to convert its state from homeostatic (M0) to reactive (M1) and inhibitory (M2) forms. In these various conditions, the effects of EVs can be stable or change. This can be due to various factors mostly contained in EVs, such as miRNAs targeted to neurons, variable in qualities and quantities, and agents secreted by involved cells, including TNFa, interleukin 1A (IL-1A) and various chemokines [33, 34].

Neuroinflammations of microglia can be affected by the concomitant activation of astrocytes. Their EVs can in fact attenuate or reinforce the microglial actions [41]. Finally, interesting exosomes and ectosomes from immune cells are investigated to improve and engineer their properties, relevant for the desired therapeutic outcomes [45]. Relevant functions of various EVs include the immunological definition of diseases. Although widely accepted and operative, such definition cannot be considered exclusive. For example, when considered from the mechanistic point of view, immunological diseases are often defined neurodegenerative, i.e. showing properties common to the other diseases.

### Encephalomyelitis

Encephalomyelitis is a severe form of brain disease. Its mechanism of generation, autoimmunity, is often employed experimentally in the mouse for peripheral induction of the multiple sclerosis (MS) disease. Various treatments and procedures have been used to induce the experimental autoimmune encephalomyelitis (EAE), not only peripherally but also in the central nervous system (CNS). In the latter, EAE can be induced by administration of interleukin-6 (IL-6), a pleiotropic and multifunctional cytokine. Interestingly, IL-6 is secreted not only by neurons, but also by astrocytes and microglia [46].

Immune responses, induced by activation of microglia together with macrophages, are rapidly followed by intense secretion of EVs. Regulation of the latter depends on cell responses. Interferon-γ (IFN-γ) and anti-inflammatory interleukin-4 (IL-4) play coordinated roles [47], valid when EVs are administered from outside the cells [48]. Astrocyte activation by IFN-γ affects the IL-6 activation of microglia, with ensuing reduction of brain EV secretion and progressive development of autoimmune encephalomyelitis [49].

Distinct results were obtained when tissues of mice treated for EAE were exposed to EVs from human mesenchymal stem cells pre-activated by IFN-γ [50, 51]. Upon a series of experiments, it was concluded that the used EVs protect multiple anti-inflammatory and neuroprotective cells [50]. Analogous results have been obtained by mouse intravenous administration of EVs secreted by human adipose mesenchymal stem cells. These treatments attenuate the proliferative potency of T cells, leukocyte infiltration and deviation [51]. The effects of EVs were found to depend on their expression of miRNAs modulated during EAE treatment. More detailed studies of EVs have identified eleven miRNAs of relevance, six protective and the other five dangerous for neurons [52, 53].

Studies of autoimmune encephalomyelitis often include the development of specific therapies. Already 10 years ago, agents such as curcumin and the Stat3 transcription inhibitor, when encapsulated within appropriate exosomes, had been shown to induce efficient anti-inflammatory effects when administered in the nasal region. From the latter site, the EVs are transferred to the brain where the drugs, taken up by microglia, induce their apoptosis [54]. However, the nasal site of administration is not as convenient as the intra-hippocampal transplantation or the retro-orbital vein injection [55]. Moreover, intravenously administered EVs generated from the human mesenchymal stem cells [50, 51] are able to alleviate the encephalomyelitis by improving its motor deficit, reducing brain atrophy, and increasing cell proliferation with decreased inflammatory infiltrations [56]. The therapy of auto-encephalomyelitis, based on miRNAs from EV cargos, is able to reduce the severity of experimental conditions, while other miRNAs induce therapeutic effects only upon their engineering [53].

### Multiple sclerosis

The prevalent inflammatory, demyelinating and neurodegenerative processes are due to immune mechanisms influenced by genetic and environmental factors. The pathology is initiated and pursued by multiple, fully or partially reversible episodes of plaque-like sclerosis, disseminated in regions of the brain, brainstem, and spinal cord. In such episodes, the loss and atrophy of neurons can be followed by irreversible neurological disability. Lesions depend on brain infiltration by immune cells, lymphocytes and myeloid cells, interactive with glial cells [57, 58]. Astrocytes alter the local gene expression of granulocyte-macrophage colonies, with increase of pro-inflammatory transcription modules [59]. Altered gene expression occurs also in oligodendrocytes, targets of immune system attacks, where demyelination is followed by degeneration of axons [60]. Evolution of MS is variable. A small fraction of patients, affected by the progressive form (PMS) of the disease, reach severe conditions within months or a few years; in the predominant form of the disease, the relapsing-remitting form (RRMS), single episodes occur separately, often of several months [57, 58].

Evidence accumulated during the last few years has demonstrated MS to depend on extensive dysregulation of neural and glial EVs, trafficking among the cells of the brain tissue and navigating the fluids, CSF and blood plasma [61–65]. The differences between RRMS and PMS forms have been further investigated by employing neuronal and astrocyte EVs isolated from the blood of human patients. Circulation of EVs, with increased production of complement induced by astrocytes, demonstrates the severity of synaptic lesions [66]. Depending on their miRNAs, the action of EVs can be different. Most miRNAs of the RRMS forms, different from those of the PMS forms, can be relevant also for the clinical development of patients [62–64]. Expansion of granulocytes with neutrophils and monocytes, together with shrinkage of lymphocytes, are typical of RRMS patients [67].

Many MS forms are interesting for the identification of biomarkers [53, 59, 61–67], and even more for new therapies [53, 59, 67]. The attempts of targeting cytokines have been reported among inconclusive results of the field [68, 69]. In contrast, immune-ablations followed by autologous hematopoietic stem cell transplantations were found to induce durable, effective, and safe therapies, similar to those obtained by monoclonal antibodies [58]. Treatment with the fingolimod drug, considered insufficiently when administered alone, was improved by the combination with selected miRNAs [70]. Aptamer, with high affinity for myelin, is bound to the EV surface. Interaction of such EVs with the cells of an oligodendrocyte line, and also with the same cells of a mouse injected in vivo, induced significant reinforcements of oligodendroglia proliferation, re-myelination, and thus axon protection [58, 68]. The results obtained so far are encouraging, especially when applied to young, less disabled patients. Recent meta-analyses have reported marked attenuation of the RRMS disease in 70–90% of patients, and long-term cessation of the disease in two thirds of young patients [71].

Use of mesenchymal stem cell exosomes induces therapy without re-induction of self-tolerance, a complication reported also for other treatments [72]. EVs are capable to interact, and thus to co-operate, with components of axons and/or oligodendrocytes, with reduction of demyelinization [73]. The final proposal refers to T lymphocytes aggressive to MS. In this case, however, the EVs secreted by regulatory cells are inhibitory of aggressive cells [74]. In conclusion, the therapeutic attempts, started several years ago, have been used until recently, with development of treatments changing life of RRMS patients. Additional, partially distinct drugs are expected soon, hopefully with further improvement of MS patients.

### Amyotrophic lateral sclerosis (ALS): an immune/ neurodegenerative disease

**Fig. 3:**
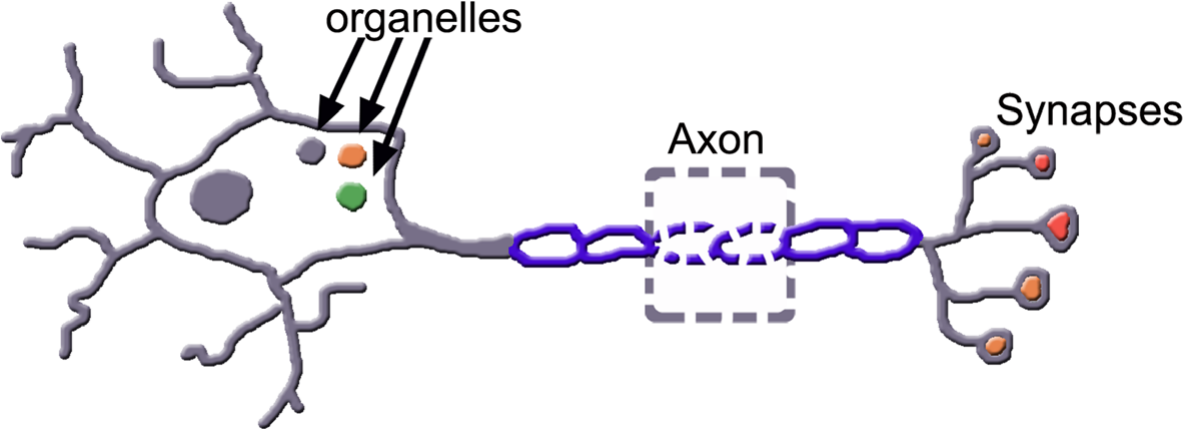
A neuron of the ALS disease shows alteration of variously distributed structures: from the cytoplasm, axon and synapses. Organelles of the cytoplasm include mitochondria (pink), lysosomes (green) and others; an axon includes two altered myelins (dashed lines) as well as several, variously altered presynaptic structures (red)

ALS is a disease of immune dysregulation affecting neuroinflammation by T lymphocytes and by proinflammatory macrophages interactive with innate immune cells, microglia and astrocytes. Such dynamic interplay becomes progressively harmful to motor neurons inducing degeneration in the motor cortex, brainstem and spinal cortex [75]. Wealth of studies demonstrated in neurons the progressive alterations of organelles and functions including mitochondria, glutamate excitotoxicity, oxidative stress, and neuroinflammation [76], accompanied by problems of axons and alteration of synapses (Fig. 3).

Recent studies aimed to the identification of misfolded proteins were carried out by comparison of mouse models, positive or not for the mutated enzyme SOD1-G93A. The observed differences, however, have been inconclusive, inappropriate for the identification of therapies effective for the disease [77]. It has therefore been hypothesized the progressive neuron alterations to depend not on specific organelles but on EVs secreted by all brain cells [76]. However, investigation of EV studies has been inconclusive. For example, recent studies [78] on EVs purified from motor neurons of patients and controls, had been carried out with postmortem tissues. The results lead to the identification of 16 distinct, differentially packaged proteins, associated with several up-regulated miRNAs. A role of EVs in ALS pathogenesis remains however hypothetical [78].

The neurodegenerative role of ALS has been investigated in relation to microglia, with EVs containing dysregulated miRNAs, destined to be transferred to neurons and thus to act as mediators of neurodegeneration [79]. The role of miRNAs has been investigated also in EVs purified from blood plasma of human ALS patients. The results obtained demonstrated that, in the EVs of human ALS, five miRNAs are overexpressed while twenty-two are low. Two such miRNAs had been previously recognized active for disability progression. The new data have now revealed processes, such as gene transcription and protein ubiquitination, affected by altered miRNAs [80].

In conclusion, the EV study of ALS has identified a number of new targets possibly important for the development of the disease. Moreover, the multiplicity of miRNAs has opened a way to continue, in the near future, the investigation of the disease. The perspectives of ALS therapy are still preliminary but promising for the near future.

## Neurodegeneration and its diseases

**Fig. 4:**
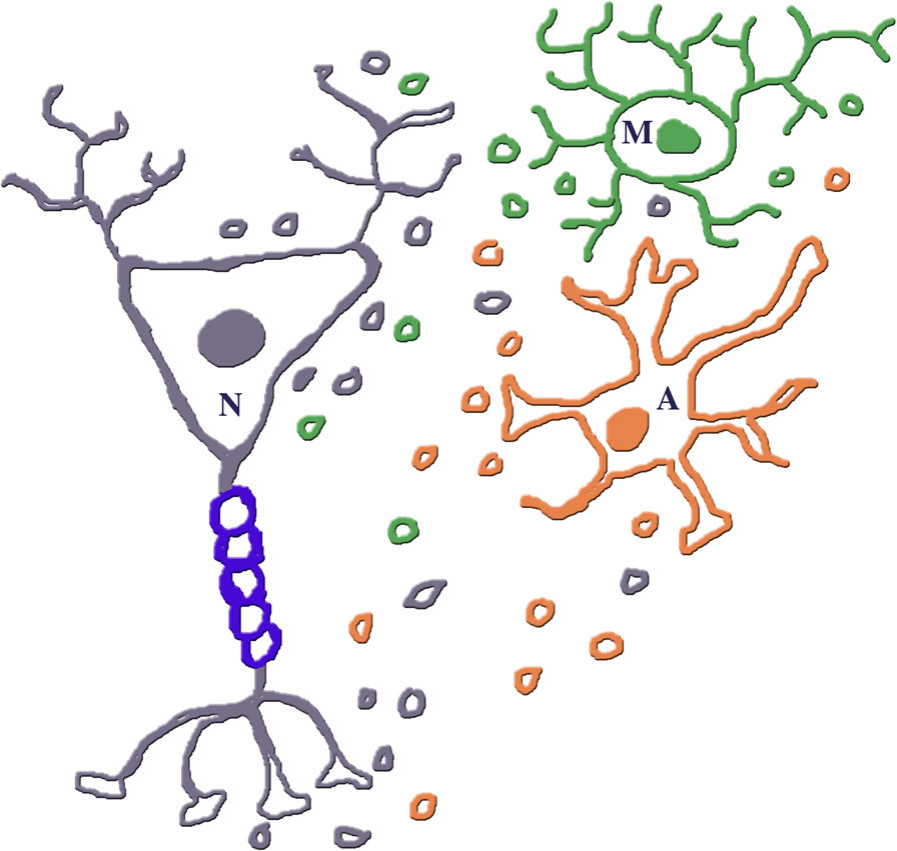
The three types of brain cells, neurons (N), astrocytes (A) and microglia (M), together with EVs they have secreted. For each parental cell and its EVs the color is the same: black for the neuron, orange for the astrocyte, green for the microglia. The blue ring sequence covering the neuronal axon is due to a myelin sheath. The EVs are distributed in the extracelluar space. Some of them are distributed close to their cell of origin, possibly because they have been secreted recently. The EV mixture of the three colors in the space among the cells may be due to vesicles addressed to specific targets, cells and fluids (CSF, blood serum, not shown)

Neurodegeneration is a pathological form that affects various brain areas. Its diseases, all characterized by specific marker proteins (such as Aβ and phosphorylated tau for AD, a-synuclein for PD, TAR43 for ALS [81]), start with local cell alterations attributed for decades to neuronal defects, expanding with time into wider lesions. At the beginning of our decade such interpretation has been corrected. Neurodegenerative diseases are now recognized to depend on the three brain cell types we have already discussed, i.e. neurons and two types of glial cell, astrocytes and microglia (Fig. 4). These cells undergo frequent interactions leading to exchange of genetic information, based either on direct surface contacts or on dynamic, highly effective forms of communication [19, 82–84]. In addition to the various forms of direct and indirect intercellular interactions, such communications are largely due to EVs, generated from and addressed to neurons, astrocytes and microglia [9, 10, 17].

Both molecular specificity and secretion of EVs are often governed by external cell stimulation. They include membranes together with many cargo molecules: multiple proteins of variable relevance; lipids; RNAs, in particular non-coding miRNAs (Fig. 1). Such molecules of EVs include properties of the cells of origin [19, 27, 34, 81–84]. Additional critical steps are developed during the onset and progression of various neurodegenerative diseases, played by the aggregation of intracellular and/or extracellular misfolded proteins [81].

The present EV review does not deal with all neurodegenerative diseases but is mostly focused on various states of the AD progress. At least some of the reported data, based on EV and miRNA studies [11, 23], are of interest also for other neurodegenerative diseases connected to AD, as shown by database-enabled analyses [85].

### Alzheimer’s disease: introduction

AD is the greatest neurodegenerative disease. It is composed by two forms: a rare genetic form that starts after a few decades of life; and a non-genetic form that increases dramatically after 60 years of age and affects millions of people throughout the world. Many reports about AD properties have already appeared (see for example [86–89]). Only a few critical aspects of the disease, discovered in neurons and glial cells, will be mentioned here. The process that induces generation of Aβ-42 (the specific peptide generated from the amyloid precursor protein (APP), from here on simply indicated Aβ), includes in sequence the cleavages by two secretases, presenilin 1 (β-secretase, BACE1) and presenilin 2 (γ-secretase). At the plasma membrane, surface Aβ binds with high affinity the prion protein PrPc, which then interacts with mGluR5, a metabotropic glutamate receptor, which in the AD conditions induces distinct responses. Another key participant of the AD pathology is tau. Such protein, largely distributed also in glial cells, plays a role in various pathologies. In AD, Aβ and hyperphosphorylated tau are widely spread together, distributed inside and outside the cells [90].

The complex assembly and the interactions summarized so far govern the human steps, inducing early AD signals followed years later by the development and then the maturation of the disease, composed by aggregated Aβ plaques accompanied by neurofibrillary tangles. For years, the developments were believed to involve only neurons. Changes of key ideas started with participation of glial cells. The co-interaction of neurons with astrocytes and microglia opened the way to ensuing processes [91–93]. In addition, various types of EV were recognized, transported from their original cells to their target cells, where cargo mixtures are discharged [2]. Early reports about such processes appeared about two decades ago [13, 94, 95]. During the following years, the number of EV publications grew progressively [14–16]. Recent knowledge about the role of EVs in Alzheimer’s [96, 97] has been chosen as a main focus of the present review. The data presented concern primarily the relevant EV details identified recently.

### Role of EVs in AD pathology

The analysis of the EVs generated by their secretion and ensuing intermixing with the various brain cells has revealed multiple mechanisms involved in AD. Occurrence of Aβ and other proteins in the EV cargos have been shown to affect various processes including synaptic function, insulin resistance, neuroinflammation, and others [98, 99]. Recent studies have shown these and other participations to be controlled by detailed mechanisms. Many such studies have been possible by the use of innovative procedures for isolation and interaction of brain EVs, distinguishing their origin from human and mouse [28].

Fusion of EVs with specific cargo proteins has been found to modify the AD cell development due to the accumulation of pathological proteins, such as Aβ and tau. In some cases, however, the peptides resulting from APP cleavage, including Aβ, can be efficiently removed from neurons, with decreased impact on neurotoxicity [100, 101]. In contrast, EV cargos containing high levels of Aβ together with phosphorylated tau can be unable to affect the long-term development of AD. Rather, they induce EV accumulation of aggressive factors such as complement and specific AD proteins [102–104].

Additional studies have demonstrated that changes of nature can be induced by AD in neurons, and also in glial cells. Stimulation of AD long-term development can be affected by aggressive mutations of tau induced by EVs in various conditions of toxicity: with involvement of EVs generated from pluripotent stem cells [105]; with EVs containing mutations of human tau that results toxic when injected into the mouse brain [106]; with tau isolated from astrocyte EV components and then accumulated by neurons [107]. Various effects reducing the AD development can be mediated by specific miRNAs, some of the non-coding RNAs that are common components of EV cargos. Two papers have documented effects of this type, both occurring in mouse AD models. In the first, miRNA-146a of EV astrocytes has been shown to restore various types of AD symptoms [108]; in the second, the effects of miR-29 are primarily of therapeutic nature [109]. Over-expression of a third, miR-340, has been shown to reduce the activity of presenilin 1, the most important enzyme for the generation of Aβ. The excess of miR-340 in brain cells reduces therefore the AD development [110].

### Biomarkers

Biomarkers are specific molecular indicators, useful for the recognition of diseases and related processes [111, 112]. The interest about biomarkers is considerable especially for biomedical approaches of AD and other neurodegenerative diseases. In addition, it also refers to distinct non-brain diseases, such as cancers, heart and others. These considerations emphasize the relevance of biomarkers and their technological improvements.

Biomarkers can be of interest in various cell conditions, such as the multiplicity of heterogeneous molecules operative in a disease; their changes occurring during development; the optimization of clinical trials; the evaluation of new drugs. AD is one of the diseases in which the study of biomarkers is intense. Simple examples are those of Aβ and hyperphosphorylated tau, two proteins concentrated in most brain areas. Many other AD biomarkers, of amyloid and non-amyloid type, have been identified working, however, not directly on the brain tissue but on EVs transferred to organ fluids, CSF and blood plasma. In order to operate their functions, biomarkers need reaching these fluids. CSF, strictly connected to extracellular fluids, is reached rapidly and therefore is an advantageous candidate for EV investigation. Blood plasma depends on the EV property to navigate across the blood-brain barrier [113], a structure precluded to drugs, antibodies and other molecules. In AD patients, the generation of EVs from brain cells is predominant, while the EV level in the CSF and, even more, in blood plasma is lower. Therefore, the evidence resulting from EV level studies, including clinical validity of derived biomarkers, can be elusive [111, 114].

Biomarkers generated from EVs are highly interesting. They are employed for the identification of many mechanisms such as those of AD and other neurodegenerative diseases [115], including the nature and the role of miRNAs contained within EVs [116, 117]. In addition biomarkers can be used to reveal various stages of a disease, including the early stages of AD, for which clinical symptoms are often unclear [118]. Diagnoses by biomarkers can activate fully prognostic evaluations. In addition to the diagnosis discussed here, many biomarkers operate also by therapeutic approaches, specific for various diseases [119, 120].

### Therapy

AD therapy has been among the most intense, and unsuccessful, medical attempts of the last decades. The use of classical receptor blockers, employed without success for many years, has been largely abandoned. More recently, attempts have been mostly based on the inhibition of APP cleavage by inhibitors of presenilin 1, a step essential for Aβ generation. Such treatment, which delays the progression of initial forms of AD, has been ineffective on developed forms of the disease. Thus it is of no interest for most patients [87]. In the last few years, attention has been focused especially on drug development, at present under investigation [121, 122]. In parallel, perspectives have been raised based on the identification of circuits and key regulators in AD cortical areas of the brain, useful for the identification of protective drugs. The action of these drugs at various stages of AD remains however to be established [123]. During the last few years the most intense studies have been based on the participation of EVs, often those secreted by mesenchymal stem cells [50, 51, 124, 125]. Such EVs, delivered intranasally or by cerebral injection, have been found to reduce the intracellular density of Aβ, inducing concomitantly immunomodulatory and neuroprotective effects [126, 127]. With other treatments, valid therapeutic effects have been reported, including export of donor properties, minimal immunogenicity, and delivery of protective genes such those encoding for neurotrophic and other factors [128, 129]. Various EVs appear therefore to offer innovative therapeutic opportunities, to be further developed by future research.

## Conclusion

The existence of EVs in the brain was discovered 20 years ago. In previous microscopic images the occurrence of small vesicles within the extracellular space had been attributed to defects of fixation. Only upon their discovery, the importance of EVs has been recognized. Based on the specific components of their membrane and, even more, of their cargos, the EVs are known to run a number of functions, with multiple, often opposite results. The contribution of these vesicles is therefore of relevance, with possible developments expected for the near future.

EVs secreted by brain cells are recognized of importance not only because of their properties: high number, extraordinary multiplicity of their active molecules, transfer from and to various cells, distribution not only in the space around the cells, the extracellular space, but also in fluids as far as the blood plasma. For quite some time the study of EVs has been oriented to cell biology and physiology, demonstrating key roles in cell life and in direct interactions among cells, close to each other (Fig. 2) and also far away. At present, the EVs, tools for the introduction of innovative processes, are important especially for brain diseases such as the immunological and neurodegenerative diseases. It should be mentioned, however, that other important brain diseases exist, including traumatic brain and spinal cord injuries, stroke, epilepsy, viral diseases, cancer (gliomas) [130–132]. Moreover, additional brain diseases are due to genetic defects such as those of miRNAs [131]. In all these [130–133] and other additional diseases, EVs play key roles analogous to those described in the present review. For most brain diseases, functional mechanisms are known. In some cases, however, important details are still unclear or even unknown Thus, the state of therapy is variable. In MS, progress during the last decades has been extraordinary [68–71]. At present, many RRMS patients look just healthy. In contrast, knowledge about AD therapy is simply frustrating. Efforts in the field, economic and scientific, have been considerable. However, results solid for millions of mature patients affected by AD have failed to emerge. At the moment perspectives exist only for early stages of the disease [108–110, 121, 122].

Based on the problems and considerations discussed in this review, significant progress is expected for the near future. EVs are one of the tools available for improvement of disease patients. Operatively, they offer windows into altered cellular and tissue states. Moreover, their detection in biological fluids potentially offers multicomponent diagnostic readouts. Moreover, the efficient exchange of cellular components can support applied use by EV-based therapeutics [133, 134]. Perspectives, promising especially for clinical developments, would be nice for all AD patients, or at least for the early fraction for which results appear to delay the development and may also induce the preventions of diseases.

## Data Availability

Data and materials were provided by the PubMed.gov Program, from the US National Library of Medicine.

## Abbreviations

Aβ: Amyloid beta-42
AD: Alzheimer’s disease
ALS: Amyotrophic lateral sclerosis
APP: Amyloid precursor protein
BBB: Blood-brain barrier
CNS: Central nervous system
CSF: Cerebrospinal fluid
EAE: Experimental autoimmune encephalomyelitis
EV: Extracellular vesicle
ICAM-1: Intercellular adhesion molecule 1
IFN-γ: Interferon gamma
IL-1A, -4 and -6: Three types of interleukin
M0, M1 and M2: Homeostatic reactive and inhibitory cells (microglia)
MS: Multiple Sclerosis
MVB: Multivesicular body
PMS and RRMS: Progressive and relapsing-remitting forms of MS
